# Diabetic-ketoacidosis in a nine-year-old child with homozygous sickle cell anaemia: a rare case report

**DOI:** 10.11604/pamj.2019.33.7.18971

**Published:** 2019-05-07

**Authors:** Nuraddeen Ibrahim, Abubakar Sani Lugga, Olayinka Rasheed Ibrahim

**Affiliations:** 1 Department of Paediatrics, Federal Medical Centre, Katsina State, Nigeria

**Keywords:** Type I diabetes mellitus, sickle cell anaemia, diabetic ketoacidosis, mesenteric crisis, child

## Abstract

Sickle cell anaemia (SCA) and type 1 diabetes mellitus (type 1 DM) are chronic medical conditions whose co-existence is uncommon in childhood. Furthermore, complications of SCA such as mesenteric crisis typically present with abdominal pain, which is also common in children with diabetic ketoacidosis (DKA) and this may possess diagnostic challenge. Herewith in, we report a rare case of a nine-year-old child with homozygous sickle cell anaemia, who presented with features of mesenteric crisis and diabetic ketoacidosis. The DKA was diagnosed based on the presence of hyperglycaemia (32.2 mmol/L), ketonaemia (4.6 mmol/L) and acidosis (11.6 mmol/L). The fluids deficit was corrected over 24 hours, with improvement in the vaso-occlusive crises (VOC) without precipitating cerebral oedema.

## Introduction

Sickle cell disease (SCD) is the most common inherited haemoglobinopathy with highest allele frequency seen in sub-Saharan Africa, Middle East and India [1]. In Nigeria, a prevalence of 2.4% was reported among children from population-based estimate while a far higher value of 11% was documented from hospital-based study [2, 3]. Sickle cell anaemia (SCA), a form of SCD affects all the organs and systems in the human body including the endocrine. Indeed, the endocrine dysfunctions appeared to be one of the most common of organ damage seen in patients with SCA [4]. This is partly attributed to the sequestration and impaction in different organs as well as iron induced cellular oxidative damage [4]. The most common endocrine disorders seen include growth delay, osteopenia and hypogonadism while diabetes mellitus (DM), thyroid and adrenal disorders are seen with less frequencies [5]. Despite the involvement of the various endocrine organs, co-existence of type 1 DM in children with SCA is very rare. Indeed, early efforts to document co-existence of type I diabetes mellitus with SCD yield extremely low prevalence, with some researchers reporting none [6]. A very few cases were reported from Nigeria among adolescents, supporting its unusual occurrence in childhood [7, 8]. Herewith in, we report a rare case of a nine-year-old pre-adolescent boy, known homozygous SCA who presented with features of mesenteric vaso-occulusive crisis (VOC) and Type I DM with diabetes ketoacidosis (DKA).

## Patient and observation

A nine-year-old male child known homozygous (HbSS), diagnosed in infancy, presented at the Emergency Paediatrics unit of the hospital with a two weeks history of polyuria and polydipsia; four days history of abdominal pain, abdominal distension and constipation. There was no family history of diabetes mellitus. Examination findings revealed a severe level of dehydration, respiratory distress (Kussmaul's breathing), subnormal axillary temperature of 35.4^°^C, and mild pallor with oxygen saturation of 92% (Finger pulse oximetry). He had abdominal distension with mild generalised tenderness and reduced bowel sounds. Other systemic examinations findings were unremarkable. Anthropometric indices were low for age; the weight of 21kg (less than the fifth percentile), a height of 127cm (12.5^th^ percentile), body mass index of 13kg/m^2^ (less than the fifth percentile) and body surface area of 0.86m^2^. Random blood glucose at presentation was 32.2 mmol/L (580mg/dL) and urine was positive for glucose, ketone and nitrite (Table 1). The admitting total WBC were 17.8 x 10^9^/L, neutrophils 71.8%, lymphocytes 22.4%, monocytes 2.9%, eosinophils 0.7%, basophils 2.2%, platelets 154 x 10^9^/L and Hb of 7.4 g/dL. Urine culture was sterile after 24hrs of incubation. He received a bolus of 0.9% normal saline at 20ml/kg over one hour and subsequently rehydrated over 24 hours (half of the total deficit given in the first eight hours and the remaining deficit with maintenance over subsequent 16 hours). Soluble insulin was commenced after the second hour at rate of 0.1unit/kg/min and later reduced to 0.05 units/kg/min due to precipitous fall in glucose. The co-existing VOC crisis was managed with nasogastric tube drainage of the stomach and analgesia and resolved after 72 hours of admission. Intravenous Ceftriaxone was given to treat precipitating urinary tract infection. Acidosis resolved after 36 hours and insulin was changed to subcutaneous premixed insulin (70/30) and discharged on 1 unit/kg/day after 12days on admission. At the follow up a week later, the self-monitoring blood glucose record ranges from 3.5 to 16mmo/L in a stable state.

**Table 1:** Laboratory findings on the day of admission

Parameter	Result	Comment
Blood glucose	32.2 mmol/L	Hyperglycaemia
Glucose in urine	2+	Glycosuria
Ketone in urine	2+	Ketonuria
Blood in urine	2+	Microscopic haematuria
Nitrite in urine	Positive	Urea splitting bacteria
Blood ketone	4.6 mmol/L	Ketonaemia
Serum bicarbonate	11.7 mmol/L	Moderate acidosis
Serum creatinine	125 µmol/L	Slightly elevated
Serum potassium	5.6 mmol/L	Elevated
Chloride	94 mmol/L	Reduced
Anion gap	26.9	Increased
Corrected serum sodium	137 mmol/L	Normal
Effective osmolality	318 mOsmol/Kg	Elevated

## Discussion

In this child, diagnosis of DKA was based on the presence of hyperglycaemia, ketonaemia (beta-hydroxybutyrate 4.6 mmol/L) and acidosis (bicarbonate 11.6 mmol/L) [9]. Besides the features of DKA, the child also presented with mesenteric VOC. The choice of fluid therapy and duration of administration presented a dilemma. The patient had severe dehydration with a new diagnosis of DKA, in which cautious rehydration is advocated because of risk of cerebral oedema [9]. In contrast, the vaso-occlusive crisis requires hyperhydration for quick resolution of signs and symptoms. The choice of fluid replacement over 24 hours successfully corrected the dehydration and treated the vaso-occlusive crises without precipitating cerebral oedema. This may be a useful alternative to the 48hrs correction recommended in children with DKA and vaso-occlusive crisis [9]. There is no convincing explanation for the rare coexistence of SCA and diabetes mellitus [10]. The short life expectancy of patients with SCA was proposed as a plausible reason. However, the prevalence of diabetes among SCD patients in Bahrain where mild haplotype allowed survival to adulthood showed lower rate compared to the general population [11]. Furthermore, despite the increasing survival to adolescence and adulthood reported among SCD cohorts, no apparent increase in prevalence of DM is seen in this population [12]. Other reasons adduced for the low prevalence included the possibility of protective roles of SCD against DM due to hyper metabolic state, low BMI and genetic factors due to proximity of β-globin gene and insulin gene on the short arm of chromosome [4, 11, 13, 14].

## Conclusion

Although not common, DKA can co-exist with VOC in child with a SCD and may possess challenge to fluid therapy. Careful rehydration over a period of 24 hours may be helpful in SCA with VOC and DKA.

## Competing interests

The authors declare no competing interests.
